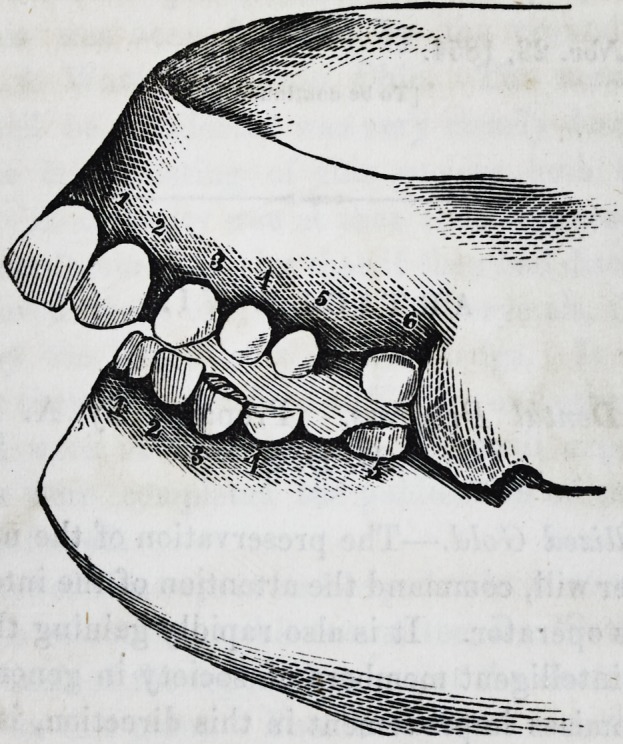# Causes of Dental Deformities, with Other Incidental Phenomena, Depending on Abnormal States of the Formative Process during Dentition

**Published:** 1855-04

**Authors:** J. L. Levison


					ARTICLE Y.
Causes of Dental Deformities, with other incidental Phenomena,
depending on Abnormal States of the Formative Process
during Dentition.
By J. L. Levison, D. D. S., &c. &c.
It will be remembered by the readers of the Quarterly Jour-
nal of Dental Science, that in the October number, for 1852,
there was among the selected articles, from the London Lancet,
one of mine, entitled "The Jaw3 and Teeth of semi-barbarous
races of men," the object of which was not so much to state an
acknowledged fact?that the jaws of civilized men are more
contracted than those of semi-barbarous races, but to prove that
this result was owing to a direct violation of the Creator's laws,
who willed that the brain and nervous system of the growing
child should not be overtaxed, and that the dental process of
attempting to build up the organic instruments and cultivate
the mental faculties at the same time, is a matter almost im-
possible to accomplish. We cannot serve two masters, if the
organic laws essential to develop the vegetative functions are to
be energetic and do their labor perfectly, the organs must not
be deprived of the vis nervosa, so essential to give tone and ac-
tivity to those important instruments of health and bodily
vigor: and it is just because this vis nervosa is too early appro-
priated by the cerebral organs (by which it is elaborated) for
the purpose of systematic mental culture, that the functions of
digestion and assimilation are interfered with, and the various
19*
224 Levison on Dental Deformities. [April,
diseases of civilized life induced; such as rickets, worms,
weak digestion, feebleness of mind and body, accompanied with
irritable nervous systems, and last not least, bad teeth, with
contracted and ill-formed jaws. The disadvantages consequent
on the latter specified injury will be rendered obvious as we
proceed. We will now merely mention that the dentist is a
new creation, being one of the few results consequent upon a
state of civilization. Some wag may say, that few dental ope-
rations are agreeable, even if the professor be ever so civil.
But let not such misunderstand my exact meaning. It is my
object to prove, that in a semi-barbarous state you have fine
and amply developed jaws, the teeth ranged in the most sym-
metrical manner?with good stamina, and hence white, beauti-
ful and durable to an old age. That these results are owing to
the active exercise such indulge in, as soon as the limbs can
move, and the incipient man or woman, begin to feel their own
volition. Thus they wanton in the delight of mere physical ex-
istence?exercising their bodies, breathing the sweet pure air?
oxygenizing their blood?they grow strong, and all the organic
functions of vegetative existence receive their highest develop-
ment. They are not set to learn lessons in symbols which only
puzzle and annoy?they read something of the book of nature,
and conserve their health by an instinctive appreciation of the
laws of their being. It is true that the savage may err in one
particular, as an animal he may eat to repletion, but then it is
in manhood. In the infancy and childhood of such semi-barbaric
nations they have such simple diet, and such constant exercise,
that they digest rapidly. It is by neglecting these lessons at
the period of development, that civilized man pays the penalty.
If with his superior knowledge of man's organism and the con-
stitution of the human mind, he would first render the body of
the child strong, and avoid any artificial methods of mental
training until the seventh or eighth year, much misery would be
spared. And as I contended in my first paper?that even with-
out any ABC instruction, the healthy infant and child, would
still be acquiring knowledge?his sight, taste, smell, touch and
hearing, acting with his perceptive powers would all be vigorous
1855.] Levison on Dental Deformities. 225
and active, and he would learn without seeming to learn, and
retain the vivid impressions which he had acquired by actual
observation and experience.
It was, therefore, from no wish to speculate on the philoso-
phy of health, when I traced to the violation of certain laws of
our corporal frame, the deformities of the mouth. The ten-
dency consisting in a contraction of the palatine bones (os pa-
lati) during the development of the teeth and alveolar processes.
This opinion is verified by the two subjoined sketches, in which
the palate presented at the union of the bones a sharp acute
angle, giving the roof the appearance of a Gothic arch, instead
of the fine flowing Saxon arch which these bones assume in
their normal condition.
In this case the temporary teeth had been removed. The defor-
mity was greatly corrected by applying a plate within the mouth
to prevent the lateral incisors from being forced out of their
and active, and he would learn without seeming to learn, and
retain the vivid impressions which he had acquired by actual
observation and experience.
It was, therefore, from no wish to speculate on the philoso-
phy of health, when I traced to the violation of certain laws of
our corporal frame, the deformities of the mouth. The ten-
dency consisting in a contraction of the palatine bones (os pa-
lati) during the development of the teeth and alveolar processes.
This opinion is verified by the two subjoined sketches, in which
the palate presented at the union of the bones a sharp acute
angle, giving the roof the appearance of a Gothic arch, instead
of the fine flowing Saxon arch which these bones assume in
their normal condition.
Fig. 1.?From a plaster cast.
In this case the temporary teeth had been removed. The defor-
mity was greatly corrected by applying a plate within the mouth
to prevent the lateral incisors from being forced out of their
Fig. 1.?1 2Central incisor?3 4 Lateral incisors, occupying their then proper
position?5 6 Cuspidati forced on the external surface of the alveolar process.
226 IjEVISON on Dental Deformities. [April,
position, and another was fixed to act externally on tlie central
incisors and cuspidati, which being made of the hippopotamus
was carried up as high as the frenum of the upper lip. The
effect in this gradual pressure, caused the palatal bones to
spread and the mouth to be more normal. The boy was feeble
and about twelve years old. It is, therefore, evident that na-
ture's intention cannot be frustrated with impunity, and many
of the evils already cited can form these inferences.
The following case is a striking instance that contracted jaws
are only part of the penalty inflicted for non-attention to phy-
sical education. It is the case of a girl about twelve, with
symptoms of scrofula, but with a good formed head, rendered
irritable by her injudicious training.
She was small, thin, pale and irritable. She had been from
her earliest childhood placed in a heated school-room "to learn
her lessons!" and she had the reward, a debilitated frame and
jaded brain. How different would have been her fate had she
position, and another was fixed to act externally on tlie central
incisors and cuspidati, which being made of the hippopotamus
was carried up as high as the frenum of the upper lip. The
effect in this gradual pressure, caused the palatal bones to
spread and the mouth to be more normal. The boy was feeble
and about twelve years old. It is, therefore, evident that na-
ture's intention cannot be frustrated with impunity, and many
of the evils already cited can form these inferences.
The following case is a striking instance that contracted jaws
are only part of the penalty inflicted for non-attention to phy-
sical education. It is the case of a girl about twelve, with
symptoms of scrofula, but with a good formed head, rendered
irritable by her injudicious training.
Fig. 2.?From a plaster cast.
She was small, thin, pale and irritable. She had been from
her earliest childhood placed in a heated school-room "to learn
her lessons !" and she had the reward, a debilitated frame and
jaded brain. How different would have been her fate had she
Fig. 2.?1 Permanent central incisor, turned on its lateral side, the other
not developed. 2 2 Central incisors. 3 3 Permanent cuspidati. 4 4 Tem-
porary grinders. 6 6 The bicuspids, (the second scarcely through the sock-
ets.) 7 7 The bicuspids. 9 10 Permanent molars.
Fig. 2.?1 Permanent central incisor, turned on its lateral side, the other
not developed. 2 2 Central incisors. 3 3 Permanent cuspidati. 4 4 Tem-
porary grinders. 6 6 The bicuspids, (the second scarcely through the sock-
ets.) 7 7 The bicuspids. 9 10 Permanent molars.
1855.] Levison on Dental Deformities. 227
been allowed plenty of exercise, pure air, simple and nutritious
diet?sound sleep, with regular sponging and friction to the
skin.
Such a student, is indeed to be pitied. For taking food af-
ter the health-destroying process, converts that which would
furnish the material for normal secretions, is converted into a
poison, and then when healthy blood is thus denied for the vigor
and growth of the body?to aggravate the evil, immediately
after such meal made to con over lessons until the head aches,
and a moral nausea is experienced for these forced tasks. The
skin, before the afternoon lessons are over, becomes dry and
feverish, the temples throb, and then the poor child has another
meal, suffers from fullness and acidity, and in this condition,
pale and jaded, goes to bed,
"To sleep, perchance to dream,"
and after a restless night, to awaken in the morning still to be
haunted with the merely mechanical lessons, to which he hur-
riedly applies before breakfast, which being swallowed rather
than masticated, the poor little creature prepares for school,
when he enters with trepidation, (for his imperfect digestion will
produce by reflex action, a palpitation of the heart,) and which
increases when the pedagogue summons the class, and with an
unsteady step and paled face he totters up for the dreaded
examination.
The repetition of these evils take place day by day, and then
it is marvelled at that there should be torpid livers, weak di-
gestions, flabby muscles, and ill-formed mouths, did not the ex-
perience of the dentist give wholesale proof of the verity of these
statements.
How can the results be otherwise? For whilst all the vari-
ous organs are undergoing changes essential to their perfect
growth, and when it would be judicious to study how to realise
a normal condition, so that life may be a constant source of joy,
rather than to endure
"All the ills which flesh is heir to."
The following case is taken from a plaster cast, the young
228 Lbyison on Dental Deformities. [April,
?
lady being about seventeen, at one of the fashionable boarding
schools. She was rather tall, thin, and the muscles flabby, with
a feeble circulation, and would have been very pretty, but for
the projection of the upper teeth. These induced a drawing up
and projection of the upper lip giving her, in consequence, an
idiotic expression.
1. The space between the upper incisors and the lower teeth
is large enough to admit the fingers being put between, when
the jaws are in apposition. Her parents were healthy and
strong, but having inherited a fortune when this young lady
was a child, they determined to make her a prodigy. The teeth
are so thin that any pressure would tend to bring on active in-
flammation and consequent decay.
The next is taken from a plaster cast, and presents another
instance of abnormal growth in one organ, and a tardiness in
another. The young lady, it was said, would have a fortune of
two hundred thousand pounds, and though she looks like an
idiot, is decidedly not one, and what would lead any one to sup-
pose mental imbecility, her chin and face were always wet, and
for which the lady preceptress often scolded her. Thus my
judgment spared the poor girl, by pointing out, that as the
saliva came into the mouth, behind the upper molar teeth, it
tended to trickle down as the jaws did not meet.
Side view, showing one of the upper incisors, lateral do.,
cuspidatus, two bicuspids and one molar. Lower jaw contains
1855.] Levison on Dental Deformities. 229
one bicuspis, and two molars, although the young lady when
this cast was taken, was two and twenty years old.
My object will be answered, if this communication should in-
duce other practitioners to enter their solemn protest against
such mad outrages of poor human nature. For in both the
latter instances the young ladies had been placed at boarding
schools, and if it were asked, "had they had regular exercise?"
the reply would be "most certainly!" But if they had been
observed under this regular exercise, it would remind one of a
number of raw recruits at their first drill. They were debarred
the free pleasure of running, jumping, hopping, skipping, et
cetera, as such exercises are so very ungenteel?they might suit
vulgar children, but not ladies, and thus health and even sym-
metry are sacrificed to some false conventional notion of what is
gentility.
Then their lessons how multifarious. Scarcely time for the
one set of ideas to fructify to leave an impression, when another
and another set of lessons are to be hurriedly got over.
Whilst music, music, music, is interspersed on the pretence of
practicing for the master, and thus the.nervous system is indeed
230 Report on Dental Progress. [April,
overturned, so that for all this refinement, like the illustrious
Franklin, they soon find that "they have paid too dear for their
whistle."
Brighton, Nov. 23, 1854.
[To be continued.]

				

## Figures and Tables

**Fig. 1. f1:**
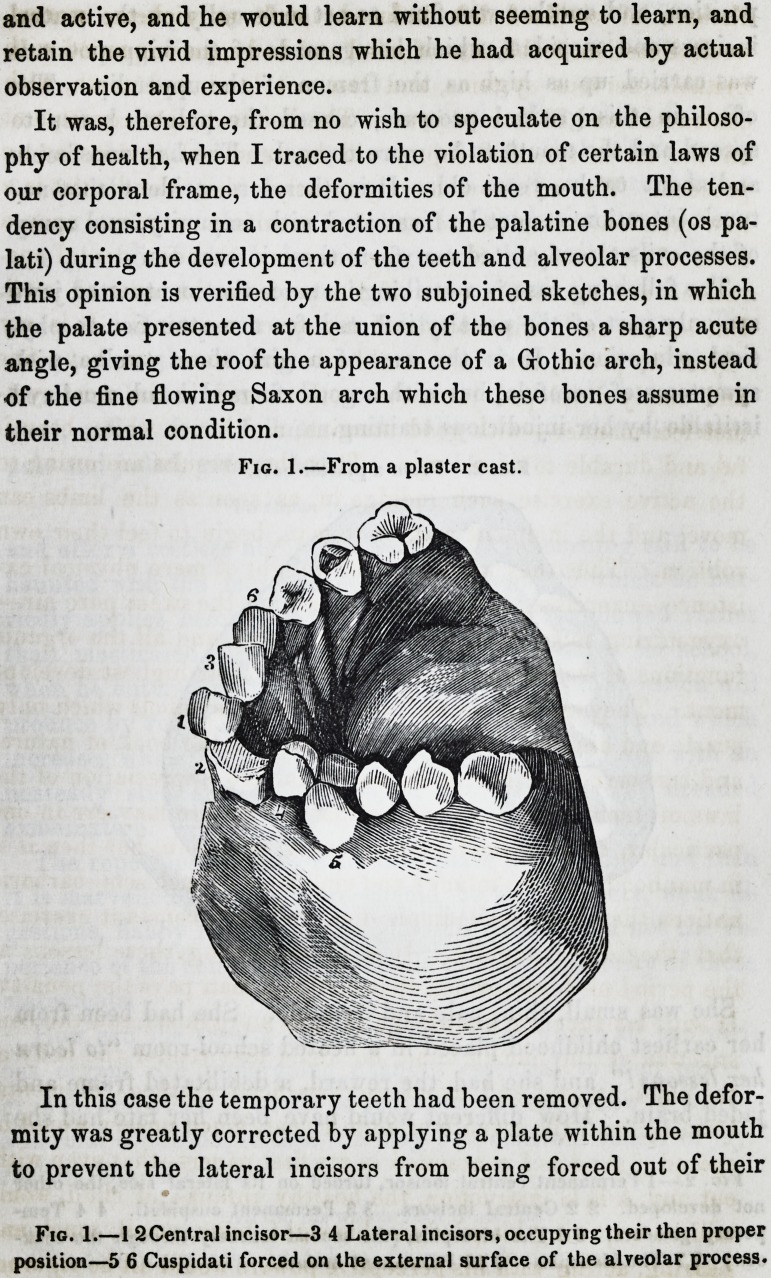


**Fig. 2. f2:**
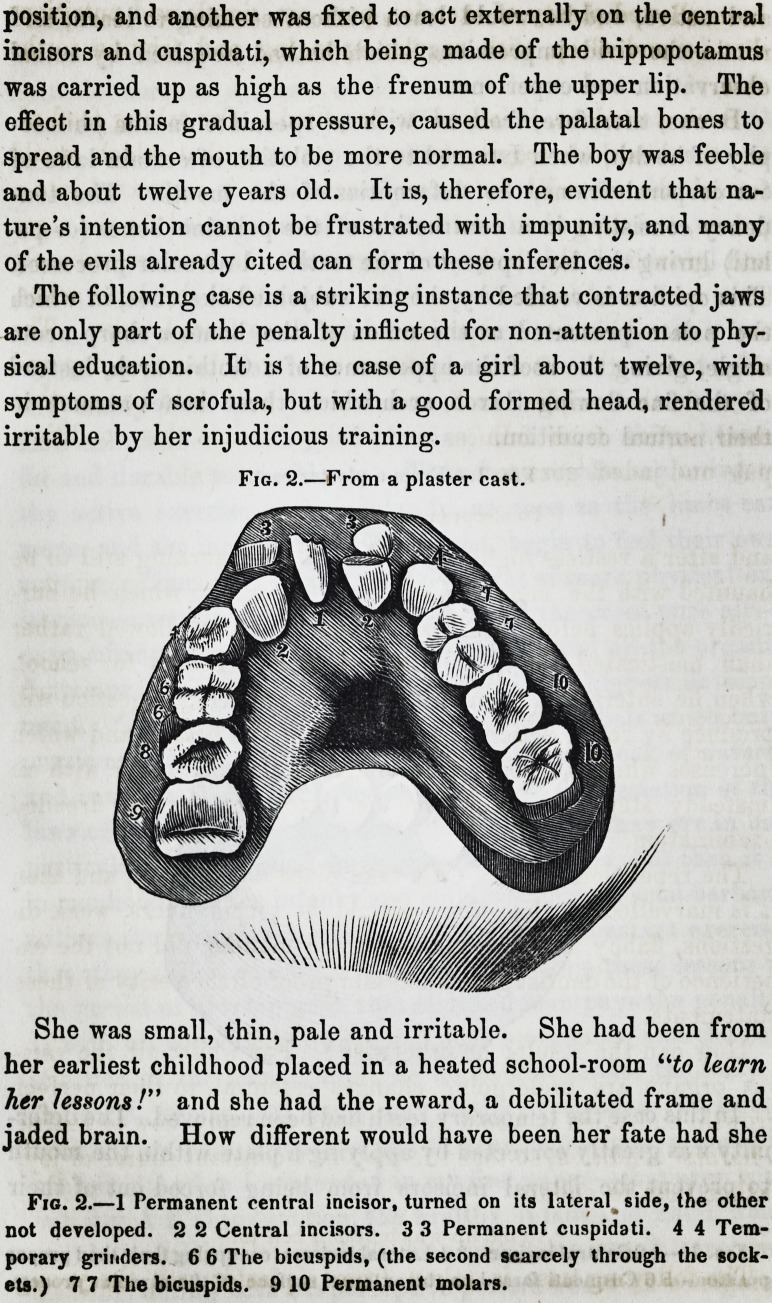


**Figure f3:**
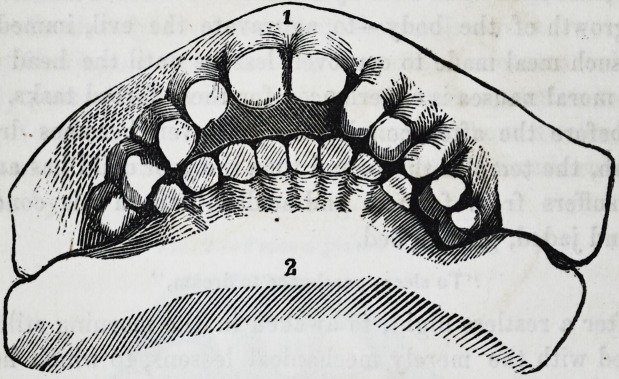


**Figure f4:**