# Errata

**Published:** 1855-10

**Authors:** 


* ERRATA.—*To the Editors.*—SIRS: In my paper on dental deformities in your April number, there are certain typographical errors, which marred the sense of the sentences in which they occur.

At line 11, page 223, the term *dental* is used instead of *dual*, which error would not have occurred had I used the word "two-fold."

At page 223, line 8, it is thus given, "and many of the evils already cited *can form*," instead of "*confirm* those inferences."

These mistakes are alluded to "more in sorrow than in anger," as it is very probable that if I did not write so small this would not occur; and the chance of error must be great in the MSS of your English correspondents, from their not seeing a proof.—*J. L. Levison's note to the editors.*

